# A Novel Risk-Scoring System to Identify the Potential Population Benefiting From Adjuvant Chemotherapy for Node-Negative TNBC Patients With Tumor Size Less Than 1 cm

**DOI:** 10.3389/fonc.2022.788883

**Published:** 2022-06-23

**Authors:** Yijun Li, Rulan Ma, Heyan Chen, Shengyu Pu, Peiling Xie, Jianjun He, Huimin Zhang

**Affiliations:** ^1^Department of Breast Surgery, The First Affiliated Hospital of Xi’an Jiaotong University, Xi’an, China; ^2^Department of Surgical Oncology, The First Affiliated Hospital of Xi’an Jiaotong University, Xi’an, China

**Keywords:** TNBC, survival, adjuvant chemotherapy, SEER, nomogram

## Abstract

**Background and Objectives:**

Whether chemotherapy is needed in node-negative triple-negative breast cancer (TNBC) patients with tumor size less than 1 cm is still controversial. In our research, we constructed a novel risk-scoring system to identify the potential TNBC patients benefiting from adjuvant chemotherapy in T1miN0M0, T1aN0M0, and T1bN0M0 stages.

**Methods:**

Relevant data were extracted from the SEER database. We applied Kaplan-Meier curves and the Cox hazards model for survival analysis and developed a nomogram of overall survival. The X-tile software was used for risk stratification. The information of TNBC patients treated in the First Affiliated Hospital of Xi’an Jiaotong University was used for the application of the model.

**Results:**

A total of 4266 patients who met the criteria of our study were included. T stage, age, race, surgery, and radiotherapy state were used to create the nomogram of overall survival. According to the total risk score, the patients were divided into high-risk (score g 73), median-risk (38 ≤ score < 73), and low-risk (score <38) groups. Chemotherapy can prolong the overall survival of patients in the median-risk and high-risk groups, while patients in the low-risk group can be exempted from chemotherapy. In addition, we also used the risk-scoring system in real-world patients as application and verification.

**Conclusion:**

We constructed a novel risk-scoring system that can be used as a chemotherapy decision-making tool for node-negative TNBC patients with tumor size less than 1 cm. Tumor size should not be the only criterion for chemotherapy treatment decision-making.

## Introduction

Triple-negative breast cancer (TNBC) refers to the lack of the expression of estrogen receptor (ER), progesterone receptor (PR), and human epidermal growth factor receptor 2 (HER2) in breast tumors. It is a special molecular subtype of breast cancer that accounts for 10%-20% of all breast cancer cases (1). TNBC has the characteristics of a distinctive metastasis pattern (2), worse prognosis (3), and more invasive biological behavior (4). Due to the lack of a therapeutic target, chemotherapy is considered to be the main method of TNBC systemic treatment.

Because of the wide implementation of ultrasound and breast X-ray examinations, an increasing number of women have been diagnosed with breast cancer at the early stage of the disease (5). Patients with the largest tumor diameter of less than 1cm are divided into T1mi (less than 1mm), T1a (1-5 mm), and T1b (5-10 mm), and usually have a good prognosis if they have negative lymph nodes and no metastasis at the same time. Whether chemotherapy is needed in TNBC patients with a tumor diameter of less than 1 cm is still controversial. Based on the National Comprehensive Cancer Network (NCCN) guidelines for BC, adjuvant chemotherapy is not recommended for T1aN0 TNBC, and could be considered for the T1bN0 subgroup. St. Gallen guidelines point out that patients with tumor size > 0.5cm could be treated with chemotherapy, while the application of adjuvant chemotherapy for T1aN0 tumors should depend on the specific situation (6). For node-negative TNBC patients with tumor size less than 1 cm, which patients would benefit from chemotherapy still needs to be further explored.

The Surveillance, Epidemiology, and End Results (SEER) database is the largest tumor database in the world and can provide abundant information about tumors and outcomes (7, 8). In our research, we conducted a retrospective study to explore the survival benefit from adjuvant chemotherapy for TNBC patients with node-negative and tumor size less than 1 cm using the SEER database.

## Materials and Methods

### Patient Selection

**Figure 1:**
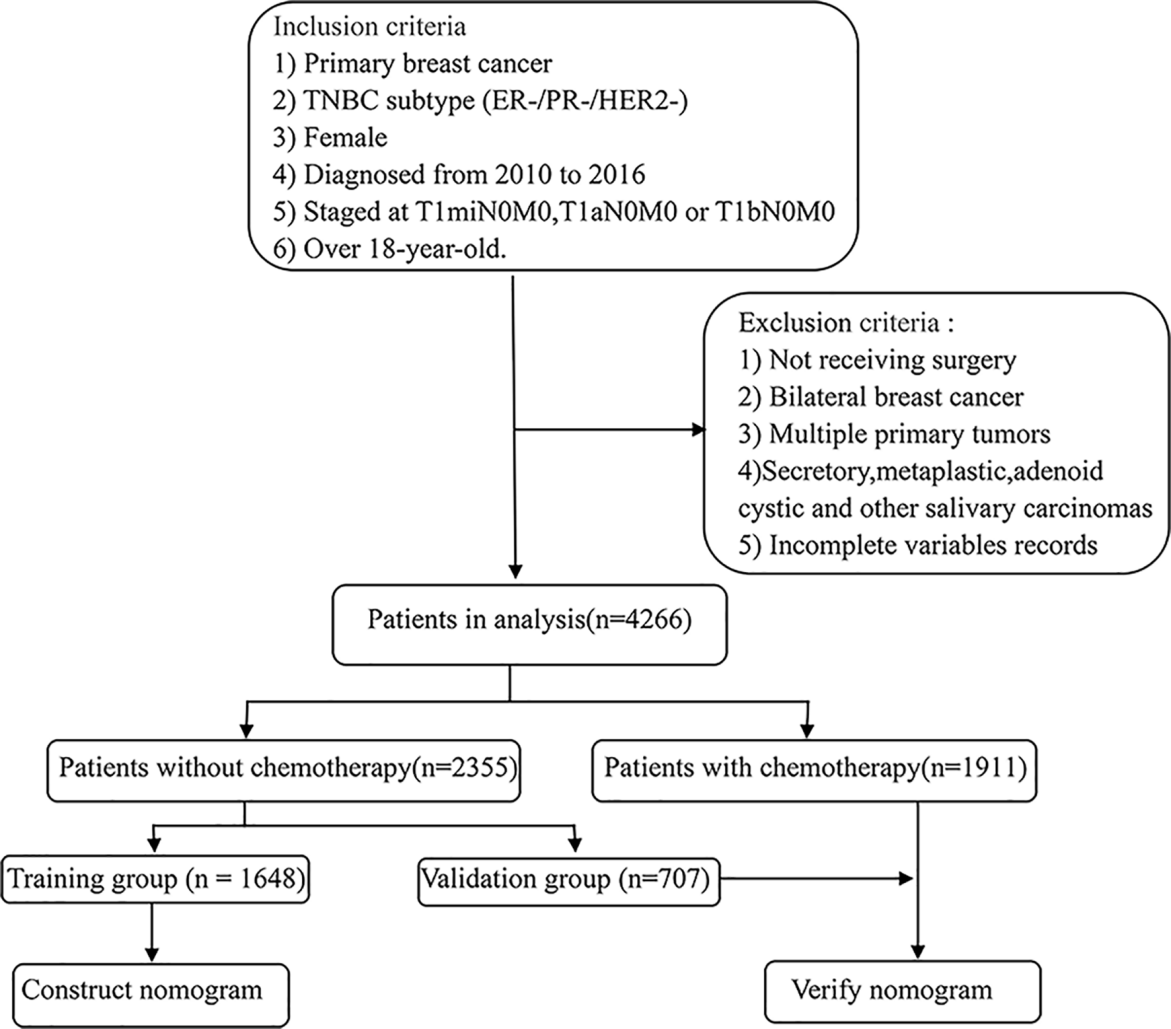
Eligibility, inclusion, and exclusion criteria of the study population. A total of 4266 patients who met the criteria were included, of which the population who did not receive chemotherapy was divided into a validation group and a training group by 1:3.

We extracted cases from the SEER database that met the following screening criteria: 1) breast cancer confirmed by histological diagnosis; 2) TNBC subtype (ER-/PR-/HER2-); 3) female; 4) years of diagnosis from 2010 to 2015; 5) staged at T1miN0M0, T1aN0M0, or T1bN0M0 based on the American Joint Committee on Cancer (AJCC) system (6th edition); 6) over 18 years old. At the same time, we excluded the following patients: 1) not receiving surgery; 2) bilateral breast cancer; 3) multiple primary tumors; 4) secretory, metaplastic, adenoid cystic, and other salivary carcinomas. Finally, we identified a total of 4266 case queues, of which the population who did not receive chemotherapy was divided into a validation group and a training group at a 1:3 ratio. The training group was used to construct nomograms, while the validation group and chemotherapy group participated in the validation of nomograms. The specific screening process is shown in **Figure 1**.

The retrospective information collection was performed on patients with T1miN0M0, T1aN0M0, or T1bN0M0 TNBC who underwent surgical treatment at the First Affiliated Hospital of Xi’an Jiaotong University from July 2015 to December 2019 for model application and verification. The specific inclusion and exclusion criteria were the same as the process of data extraction from the SEER database (**Figure 1**). The study was approved by the Ethics Committee of the First Affiliated Hospital of Xi’an Jiaotong University.

### Research Variables

The study included the following clinicopathological variables: T stage, age, race, grade, laterality, radiotherapy, chemotherapy, marital status, survival time, and survival state. Breast cancer specific survival (BCSS) is defined as the time from diagnosis to death due to breast cancer. Overall survival (OS) refers to the interval from the date of diagnosis to the date of death from any cause. Disease-free survival (DFS) is regarded as the time from diagnosis of cancer to cancer recurrence, metastasis, or death due to cancer progression.

### Statistical Analyses

We applied Pearson’s *χ*2 test to compare baseline differences in clinicopathological parameters between the non-chemotherapy and the chemotherapy groups. The Kaplan-Meier curve as well as the log-rank test were performed to compare survival differences between groups. The Cox proportional hazards model was built to predict the effect of covariates on survival outcomes. Subsequently, a nomogram was developed based on the multivariable Cox regression results, and 1000 bootstrap resampling internal verification, calibration curve, and the concordance index (c-index) were used to evaluate the accuracy and the discrimination of the model. The X-tile software was applied to determine the best cut-off value of the score. *P <*0.05 was set to define significant differences. All statistical calculations were performed in SPSS 24.0 (SPSS statistics, Chicago, IL, USA) and R 4.1.0 (R Project for Statistical Computing) software.

## Results

### Patient Characteristics

From 2010 to 2016, a total of 4266 patients who met the criteria of our study were included, of which 2355 (55.2%) received chemotherapy and 1911 (44.8%) did not. Detailed baseline information of patients is shown in **Table 1**. There are significant differences in T stage, age, race, tumor grade, marital status, surgery, and radiotherapy state between the non-chemotherapy and the chemotherapy group. Among all the population, the cases with T1b stage, younger than 50 years old, other race, III/IV grade, receiving mastectomy and radiation treatment, or married were more likely to receive adjuvant chemotherapy. No difference in tumor laterality was observed between the two cohorts.

**Table 1:** Clinical characteristics of node-negative triple-negative breast cancer patients with tumor size less than 1 cm from the SEER database.

Characteristics	Total	Non-Chemotherapy	Chemotherapy	P
N	4266	2355 (55.2)	1911 (44.8)	
T				<0.001
T1mi	201	183 (91.0)	18 (9.0)	
T1a	1181	906 (76.7)	275 (23.3)	
T1b	2884	126 6(43.9)	1618 (56.1)	
Age at diagnosis (yrs.)				<0.001
<50	620	203 (32.7)	417 (67.3)	
≥50	3646	2152 (59.0)	1494 (41.0)	
Race				<0.001
White	2953	1670 (56.6)	1283 (43.4)	
Black	602	317 (52.7)	285 (47.3)	
Other	711	368 (51.8)	343 (48.2)	
Grade				<0.001
I	247	201 (81.4)	46 (18.6)	
II	1361	899 (66.1)	462 (33.9)	
III/IV	2658	1255 (47.2)	1403 (52.8)	
Laterality				0.274
Right	2215	1205 (54.4)	1010 (45.6)	
Left	2051	1150 (56.1)	901 (43.9)	
Surgery and Radiotherapy				0.028
BCS + Radiation	2050	1104 (53.9)	946 (46.1)	
Mastectomy	1509	866 (57.4)	643 (42.6)	
BCS	633	361 (57.0)	272 (43.0)	
Mastectomy + Radiation	74	24 (32.4)	50 (67.6)	
Marriage status				<0.001
Married	2457	1267 (51.6)	1190 (48.4)	
Unmarried/DSW	1591	953 (59.9)	638 (40.1)	
Unknown	218	135 (61.9)	83 (38.1)	

BCS, breast conserving surgery;

DSW, divorced & separated & widowed.

### Chemotherapy and Survival

**Figure 2:**
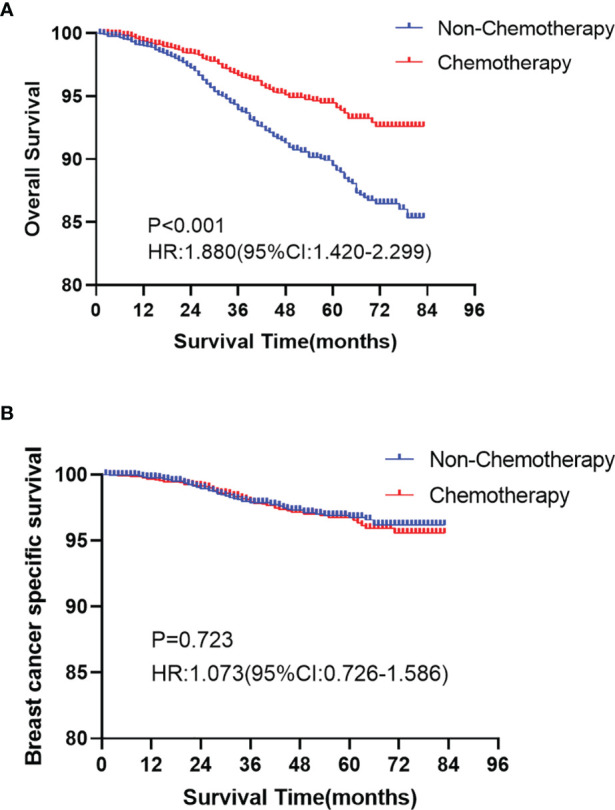
Kaplan-Meier curves analyses of the overall survival (OS, **A** *P* < 0.001) and the breast cancer specific survival (BCSS, **B** *P >*0.05) for chemotherapy and non-chemotherapy group in node-negative triple-negative breast cancer with tumor size less than 1 cm.

**Figure 3:**
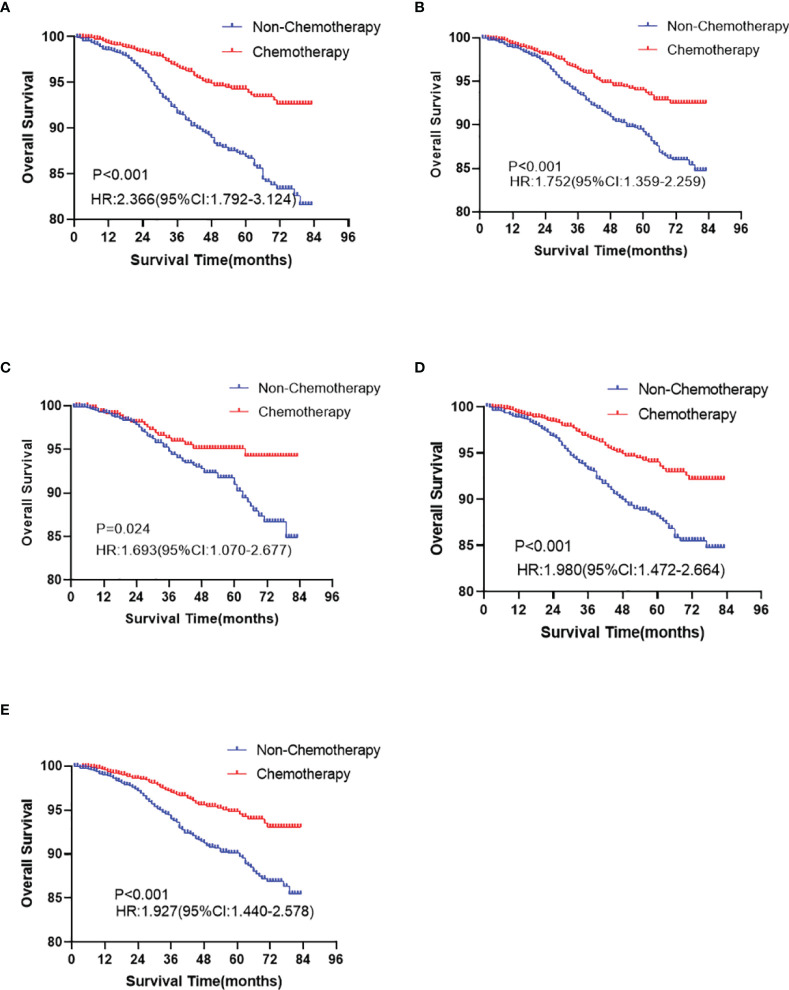
The overall survival analyses for chemotherapy and non-chemotherapy group in T1b (**A** *P* < 0.001), ≥50 years old (**B** *P* < 0.001), grade II (**C** *P* = 0.024), III/IV (**D** *P* < 0.001), and White (**E** *P* < 0.001) race subgroups in node-negative triple-negative breast cancer with tumor size less than 1 cm.

In the Kaplan-Meier curve survival analysis and the log-rank test, the OS of the chemotherapy group was significantly higher than that of the non-chemotherapy group (*P* < 0.001). The OS of 5-year rates in the chemotherapy group and the non-chemotherapy group were 94.1% vs. 89.4%, respectively (**Figure 2A**). However, no statistical difference was observed between the two groups in BCSS (*P >*0.05, **Figure 2B**). In subgroup analysis, chemotherapy can significantly prolong the OS (*P* < 0.05), which appears in T1b (**Figure 3A**), over 50 years old (**Figure 3B**), grade II (**Figure 3C**), grade III/IV (**Figure 3D**), and White race (**Figure 3E**) subgroups. However, in T1mi or T1a, younger than 50 years old, grade I, Black race, and other races subgroups, there was no significant difference in OS between chemotherapy and non-chemotherapy groups (*P >*0.05). Moreover, whether chemotherapy or not could not improve BCSS in all subgroups.

### Univariable and Multivariable Cox Proportional Hazards Model

The Cox proportional hazards model was established to identify prognostic factors of TNBC patients with node-negative and tumor size less than 1 cm (**Table 2**). The univariable analysis showed that cases with T1mi, breast conserving surgery and radiotherapy, younger than 50 years old, and other races tended to have a better OS. Multivariable analysis confirmed that T stage, age, race, surgery, and radiotherapy status were independent predictors of OS.

**Table 2:** Univariable and multivariable Cox proportional hazard model analysis of overall survival in node-negative triple-negative breast cancer patients with tumor size less than 1 cm.

Variable	Univariable analysis			Multivariable analysis		
	HR	95% CI	P	HR	95% CI	P
T			0.050			0.001
T1mi	1			1		
T1a	1.014	0.518-1.983	0.968	1.010	0.515-1.980	0.977
T1b	1.417	0.751-2.674	0.282	1.733	0.910-3.299	0.094
Age at diagnosis (yrs.)			0.003			0.029
<50	1			1		
≥50	1.921	1.242-2.971		1.647	1.053-2.577	
Race			0.006			0.016
White	1			1		
Black	1.489	1.095-2.025	0.011	1.456	1.069-1.982	1.069
Other	0.757	0.52-1.102	0.146	0.783	0.537-1.141	0.537
Grade			0.583			
I	1					
II	1.267	0.692-2.319	0.444			
III/IV	1.345	0.749-2.413	0.321			
Laterality			0.640			
Right	1					
Left	0.945	0.744-1.199				
Surgery and Radiotherapy			<0.001			<0.001
BCS + Radiation	1			1		
Mastectomy	1.531	1.163-2.015	0.002	1.631	1.236-2.152	0.001
BCS	2.032	1.463-2.822	<0.001	1.948	1.401-2.708	<0.001
Mastectomy + Radiation	2.997	1.607-5.589	0.001	3.576	1.911-6.691	<0.001

BCS, breast conserving surgery;

DSW, divorced & separated & widowed.

### Nomogram Development and Validation

**Figure 4:**
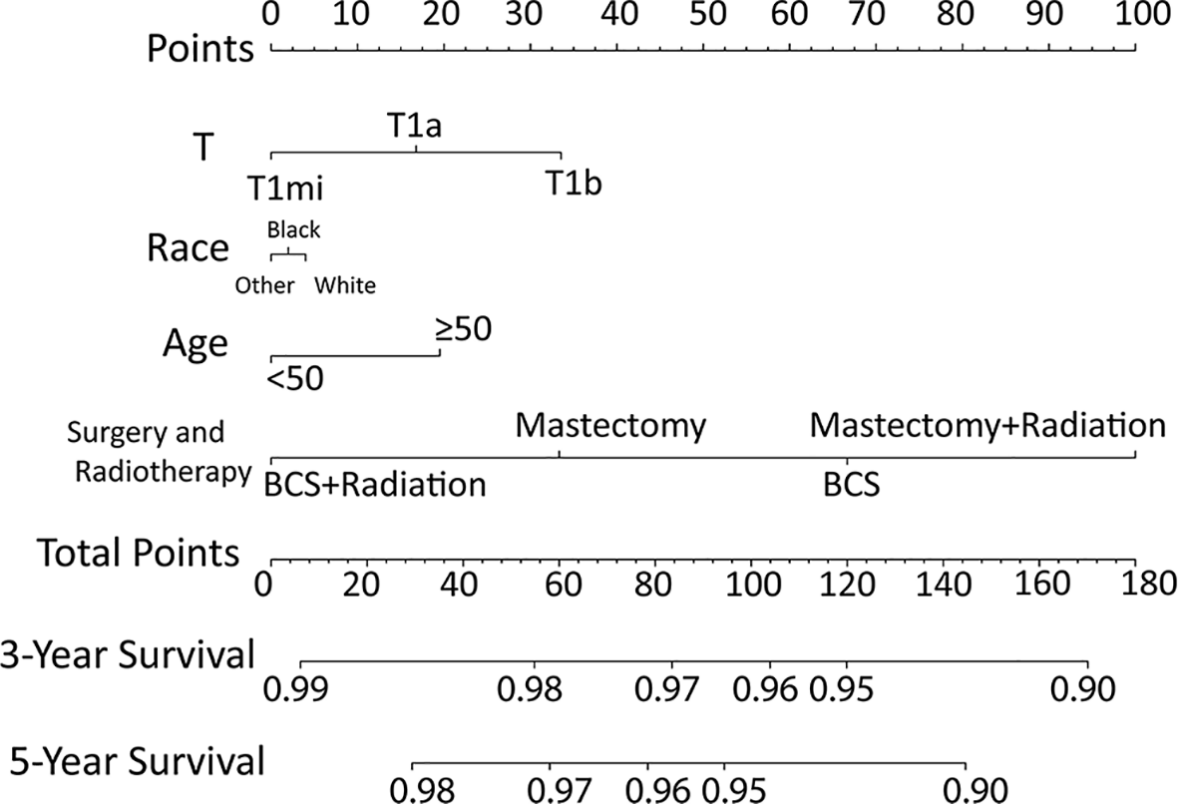
Nomogram for predicting 3‐ and 5‐year overall survival among node-negative triple-negative breast cancer patients with tumor size less than 1 cm.

**Figure 5:**
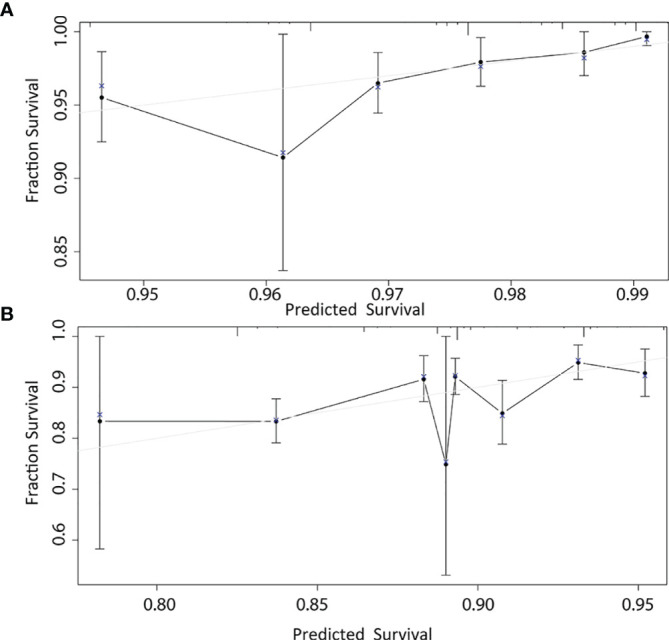
1000-bootstrap resampling internal verification correction in 3-year **(A)** and 5-year **(B)** for a nomogram of the overall survival in node-negative triple-negative breast cancer patients with tumor size less than 1 cm.

**Figure 6:**
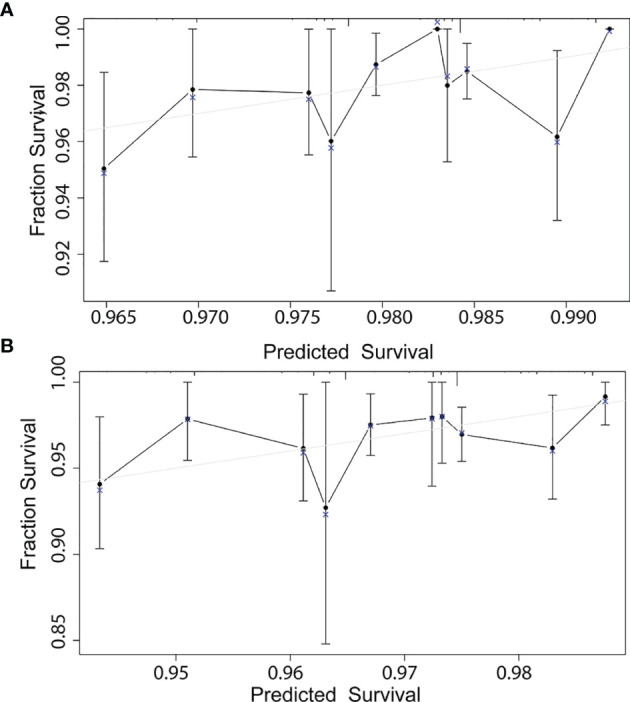
External validation correction in 3-year **(A)** and 5-year **(B)** for a nomogram of the overall survival in node-negative triple-negative breast cancer patients with tumor size less than 1 cm.

According to the results of multivariable Cox model, we established a nomogram based on independent predictors to predict the 3-year and 5-year OS (**Figure 4**) and the risk score of each independent prognostic factor is shown in **Table 3**. Subsequently, the c-index was used to measure the discrimination of the model, and the internal validation curve was drawn to evaluate the consistency. The C-index of the nomogram was 0.818 (95CI%:0.771-0.865), and the calibration curve showed the strong correlation between the estimated and the predicted value in 3-year (**Figure 5A**) and 5-year OS (**Figure 5B**). The validation group and chemotherapy group were used for external validation. The C-index of the validation cohort was 0.739 (95CI%:0.693-0.785), and there was no significant deviation between the actual OS curve of 3-year (**Figure 6A**), 5-year (**Figure 6B**), and the prediction curve, which indicated that the nomogram was also applicable to the validation set. Taken together, both the internal and external validations have proven the high predictive value of the nomogram.

**Table 3:** The risk score of each independent prognostic factor and the criteria for risk subgroup stratification.

Characteristics	Points
T	
T1mi	0
T1a	17
T1b	34
Age at diagnosis (yrs.)	
<50	0
≥50	20
Race	
White	4
Black	2
Other	0
Surgery and Radiotherapy	
BCS + Radiation	0
Mastectomy	33
BCS	67
Mastectomy + Radiation	100
Risk subgroup	
low-risk	<38
median-risk	38- 73
high-risk	≥73

BCS, breast conserving surgery.

### Benefits of Chemotherapy in Each Risk Group

**Figure 7:**
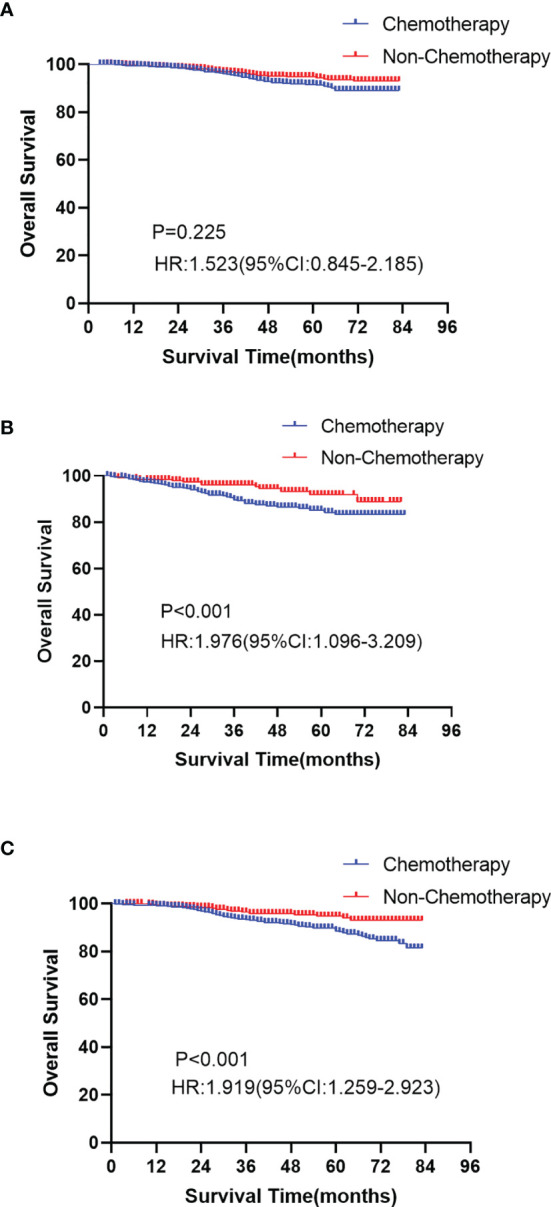
Kaplan-Meier curves of overall survival between the chemotherapy and non-chemotherapy cohort in the low-risk group (**A** *P*=0.025), median-risk group (**B** *P* < 0.001) and high-risk group (**C** *P* < 0.001).

As shown in **Table 3**, we calculated the risk score of each patient, and used X-tile software to divide the patients into high-risk (score ≥ 73), median-risk (38 ≤ score < 73), and low-risk (score <38) groups according to the total risk score. In the training group (N=1648), 502 (30.46%) patients were in the high-risk group, 891 (54.07%) patients were in the median-risk group, and 255 (15.47%) patients were in the low-risk group. In the validation group (N=2618), 889 (33.96%) patients were in the high-risk group, 1297 (49.54%) patients were in the median-risk group, and 432 (16.50%) patients were in the low-risk group. In the low-risk group, the 3-year and 5-year survival rates were 96.4% and 92.8%, respectively. In the median-risk group, the 3-year and 5-year survival rates were 94.2% and 91.2%, respectively. In the high-risk group, the 3-year and 5-year survival rates were 92.5% and 86.6%, respectively. The survival differences between the chemotherapy group and non-chemotherapy group in each risk group were compared by log-rank test. The Kaplan-Meier curve showed that patients in the low-risk group did not have prolonged OS from chemotherapy (P = 0.225, **Figure 7A**), while chemotherapy could significantly improve the OS of patients in the median-risk (P < 0.001, **Figure 7B**) and high-risk (P < 0.001, **Figure 7C**) groups. Therefore, our model successfully screened out patients who could benefit from chemotherapy.

### Application of the Risk-Scoring System

In order to display the application of the model, we retrospectively collected 35 T1miN0M0, T1aN0M0, or T1bN0M0 TNBC breast cancer patients who underwent surgery in our department from July 2015 to December 2019 (**Table 4**). According to the guideline of the Chinese Society of Clinical Oncology (CSCO), we recommended postoperative chemotherapy for all TNBC patients with small tumor, but there were still seven (20.0%) patients who didn’t receive chemotherapy due to various reason. In the low-risk group, 80.00% (4/5) of patients received chemotherapy. In the median-risk group, 77.78% (7/9) of patients received chemotherapy. In the high-risk group, 80.95% (17/21) of patients received chemotherapy. At the same time, we scored all patients according to the scoring system. The results showed that 18.6% of patients were in the low-risk group and could be exempted from chemotherapy. During our follow-up until July 2020, two patients experienced recurrence and one patient died. The specific information of recurrent and deceased patients is shown in **Table 5**. The deceased patient (number 1) and one of the recurrent patients (number 3) did not receive chemotherapy, however. According to the scoring system, these two patients belong to high-risk and medium-risk groups, respectively, and should have received adjuvant chemotherapy. Real world data show that our model can accurately identify patients who do not need chemotherapy, and provides a basis for the necessity of chemotherapy in medium- and high-risk groups.

**Table 4:** Clinical characteristics of node-negative triple-negative breast cancer patients with tumor size less than 1 cm from the First Affiliated Hospital of Xi’an Jiaotong University.

Characteristics	Number
N	35
T	
T1mi	3 (8.6)
T1a	6 (17.1)
T1b	26 (74.3)
Age at diagnosis (yrs.)	
<50	15 (42.9)
≥50	20 (57.1)
Histological grade	
I	5 (14.3)
II	8 (22.9)
III	22 (62.9)
Laterality	
Right	19 (54.3)
Left	16 (45.7)
Surgery and Radiotherapy	
BCS + Radiation	13 (37.1)
BCS	2 (5.7)
Mastectomy	20 (57.1)
Marriage status	
Married	33 (94.3)
Unmarried/DSW	2 (5.7)
Chemotherapy	
Yes	28 (80.0)
No	7 (20.0)

BCS, breast conserving surgery.

**Table 5:** Clinical characteristics of patients with death and recurrence during follow-up.

Characteristics	Patients		
	1	2	3
Vital status	Dead	Recurrence	Recurrence
DFS (months)	41	45	37
T	T1b	T1b	T1a
Age at diagnosis (yrs.)	79	42	60
Histological grade	III	III	III
Laterality	Right	Right	Left
Surgery and Radiotherapy	Mastectomy	Mastectomy	Mastectomy
Marriage status	Married	Married	Married
Chemotherapy	No	Yes	No
Risk score	87	67	70
Risk classification	High-risk	Median-risk	Median-risk

DFS, Disease-free survival.

## Discussion

At present, there are different opinions about whether TNBC patients with tumor size less than 1 cm need chemotherapy. NCCN (Version 6, 2021) guidelines recommend TNBC patients with stage T1aN0M0 do not need adjuvant chemotherapy and use the word “consideration” for T1bN0M0 patients. In the 17th St. Gallen International Breast Cancer Conference, according to the the experts’ voting results, 45.6% experts support that the appropriate tumor size threshold of lymph node-negative TNBC breast cancer for adjuvant therapy should be 5 mm. At the same time, the European Society of Oncology (ESMO) guidelines indicate that patients with TNBC should receive adjuvant chemotherapy except for low-risk T1aN0M0 tumors. Based on the guideline of the CSCO (Version 2022), all TNBC patients with T1a-bN0M0 stage should receive standard adjuvant chemotherapy (9). In the records of SEER database, more than 50% of patients in T1b received chemotherapy, and it was the highest proportion among T1mi, T1a, and T1b stage. For T1a stage patients, only 23.3% of cases were treated with chemotherapy. T1mi patients had the lowest chemotherapy acceptance rate, accounting for 9.0% of all patients. In our center, approximately 80.0% of patients received chemotherapy, which is related to different guidelines and principles in different countries. Clinicians in China are more active in the implementation of chemotherapy.

Due to the lack of prospective evidence, it is controversial whether adjuvant chemotherapy can improve the survival of TNBC patients with small tumor. In a retrospective study involving 363 cases, chemotherapy can improve DFS survival in TNBC patients, even for the T1a stage (10). Some researchers indicate that chemotherapy is not necessary for T1a-bN0M0 TNBC patients because the prognosis of T1 stage breast cancer is good enough (11–14). There is no doubt that larger tumor diameter often means greater tumor burden, therefore, the larger the tumor diameter is, the higher the risk score is generated by our model, especially for T1bN0M0 patients (15–17). However, there are many clinicopathological indicators affecting the prognosis of the tumor, including age, race, surgery, and so on. It is not accurate to determine whether chemotherapy is needed by tumor size alone. The previous studies including guidelines mostly focus on the correlation between chemotherapy and survival in TNBC patients with different stages (18, 19) which may be the reason for different research conclusions. In our study, we have successfully developed a scoring system according to tumor T stage, age, race, surgery, and radiotherapy status, which can accurately screen the people who can benefit from chemotherapy. Both internal and external validations have proven that the model has good calibration.

In addition to tumor size, age, race, surgery, and radiotherapy are also important indicators for chemotherapy decision-making. It was previously believed that younger patients had a lower OS and BCSS rate and a higher local recurrence possibility than older patients, and it also associates with the occurrence of small tumors (20, 21). In our study, we found that patients older than 50 years old had a poorer prognosis but could benefit from chemotherapy for OS. This may be related to the fact that chemotherapy can reduce non-tumor specific death in elderly patients. It has been reported that as a special subtype of breast tumor, the prognosis of TNBC is not affected by classical prognostic factors (22, 23), which is also one of the possible mechanisms.

Breast cancer incidence rate, treatment rate, and treatment sensitivity vary widely among races (24–26), which is related to genetic and socioeconomic causes (27). For example, because the average income and education level are lower than those of Whites, the breast cancer patients of the Black race are diagnosed later and have a worse prognosis. Moreover, the unique BRCA1 gene mutation found in Blacks may explain the fact that the proportion of TNBC subtypes is much higher than that of whites (27). According to the results of subgroup analysis, the White and Black races have a worse prognosis, therefore, they are more likely to benefit from chemotherapy. Whether chemotherapy is necessary for other races needs further investigation.

Previous studies suggest that the long-term survival rate of breast conserving surgery (BCS) combined with radiotherapy for early breast cancer is at least as good as that of mastectomy (28–30). However, recent studies have shown that patients with BCS combined with radiotherapy have the higher survival rate than mastectomy (31, 32). BCS may be a more appropriate method for local treatment of early breast cancer. Our model also shows that the risk of BCS+ radiotherapy is lower than mastectomy, and adjuvant chemotherapy is more needed after mastectomy. However, the potential biological mechanism of this phenomenon is still unclear. One possibility is that adjuvant radiotherapy can reduce local recurrence.

Applying our model to real-world patients, we found that 14.3% of patients belong to the low-risk group and cannot benefit from chemotherapy. However, according to the CSCO guidelines of breast cancer, we recommended adjuvant chemotherapy for all patients. This greatly increases the economic burden of patients, medical insurance pressure, and chemotherapy-related side effects. Therefore, we may consider reducing the population of chemotherapy in the low-risk group for the future CSCO guidelines. During our follow-up, the patients who died or relapsed belonged to the medium- or high-risk group, and two of these three patients refused receiving chemotherapy. For medium- and high-risk patients who are in good physical condition and can tolerate chemotherapy, the necessity of chemotherapy can be emphasized to avoid premature recurrence and metastasis leading to a prolonged treatment cycle and limited survival.

As a result of a retrospective rather than prospective study, there still exist some limitations in our research. Firstly, since the SEER database did not provide information on Her2 until 2010, the follow-up time of our cohort was short. Secondly, the SEER provides limited data, and the information like specific chemotherapy regimen, Ki-67 index, gene detection is unknown, which limits the application of nomogram. Our study still needs longer follow-up, larger sample size, and prospective investigation for further validation.

## Conclusion

We constructed a novel risk-scoring system that can be used as a chemotherapy decision-making tool to identify potential populations benefiting from adjuvant chemotherapy for node-negative TNBC patients with tumor size less than 1 cm. Chemotherapy can prolong the OS of patients in the medium-risk and high-risk groups, while patients in the low-risk group can be exempted from chemotherapy. Tumor size should not be the only criterion for chemotherapy treatment decisions.

## Data Availability Statement

The raw data supporting the conclusions of this article will be made available by the authors, without undue reservation.

## Ethics Statement

The studies involving human participants were reviewed and approved by the Ethics Committee of the First Affiliated Hospital of Xi’an Jiaotong University. Written informed consent for participation was not required for this study in accordance with the national legislation and the institutional requirements.

## Author Contributions

YL and RM designed the research. HC, PX, and SP performed the experiments. JH and HZ wrote and edited the manuscript. The authors read and approved the final manuscript.

## Funding

The research was supported by the Key Research and Development Project of Shaanxi Province (No. 2020SF-294).

## Conflict of Interest

The authors declare that the research was conducted in the absence of any commercial or financial relationships that could be construed as a potential conflict of interest.

## Publisher’s Note

All claims expressed in this article are solely those of the authors and do not necessarily represent those of their affiliated organizations, or those of the publisher, the editors and the reviewers. Any product that may be evaluated in this article, or claim that may be made by its manufacturer, is not guaranteed or endorsed by the publisher.
